# Bacteriophages as Fecal Pollution Indicators

**DOI:** 10.3390/v13061089

**Published:** 2021-06-07

**Authors:** Daniel Toribio-Avedillo, Anicet R. Blanch, Maite Muniesa, Lorena Rodríguez-Rubio

**Affiliations:** Department of Genetics, Microbiology and Statistics, University of Barcelona, Diagonal 643, 08028 Barcelona, Spain; d.toribio.avedillo@ub.edu (D.T.-A.); ablanch@ub.edu (A.R.B.); mmuniesa@ub.edu (M.M.)

**Keywords:** bacteriophages, indicator, fecal pollution

## Abstract

Bacteriophages are promising tools for the detection of fecal pollution in different environments, and particularly for viral pathogen risk assessment. Having similar morphological and biological characteristics, bacteriophages mimic the fate and transport of enteric viruses. Enteric bacteriophages, especially phages infecting *Escherichia coli* (coliphages), have been proposed as alternatives or complements to fecal indicator bacteria. Here, we provide a general overview of the potential use of enteric bacteriophages as fecal and viral indicators in different environments, as well as the available methods for their detection and enumeration, and the regulations for their application.

## 1. Introduction

Life on our planet cannot exist without water and it is estimated that 50% of the global human population lives close to rivers, lakes, or oceans [1]. Besides its importance in maintaining health and hygiene, water is also essential for economic and productive activities (such as agriculture, industry, tourism, transportation, etc.), recreation and leisure (swimming pools, fountains, etc.), and the preservation and restoration of natural ecosystems. Therefore, a decrease in water quality due to pollution poses a risk to human wellbeing and the natural environment.

One of the main sources of water pollution is the discharge of human and animal fecal waste. If poorly managed, effluents of wastewater treatment plants (WWTPs) and industrial and livestock wastes can spread enteric pathogens, including viruses, into aquatic environments. Though crucial for human health and development, the microbiological quality of water is difficult to control, due to the variety of existing waterborne pathogens, the lack of effective methods, and the analytical and logistics costs required to detect such a high number of pathogens (parasites, bacteria and viruses) [2]. The definition of indicator, index, and model microorganisms, more than a century ago, allowed such limitations to be overcome by ensuring a sufficiently appropriate control of water quality.

Despite their limitations, fecal indicator microorganisms represent a useful tool to monitor the microbiological quality of water, where their presence is a sign of fecal contamination and potentially the existence of pathogens. The most commonly used are fecal indicator bacteria (FIB), including total coliforms, fecal coliforms, *Escherichia coli*, streptococci and enterococci [3].

One of their drawbacks is that they do not provide information on the source of fecal contamination, being frequently found in the microbiota of many animals. Moreover, they correlate poorly with human viruses or parasites pathogens in natural aquatic environments and WWTPs, displaying different behavior and lower survival rates [3].

Recently, bacteriophages capable of infecting enteric bacteria have been proposed as alternative indicators of fecal and viral pollution. Bacteriophages have several advantages over bacterial indicators, as they are more abundant and generally more persistent in the environment and can provide more accurate information about viral pathogens. Bacteriophages serve as useful indicators of fecal contamination, as they are eliminated in feces, and do not replicate in a natural environment unless their host is present and metabolically active [4]. They can also indicate viral contamination, as bacteriophages infecting intestinal bacteria spread into the environment in a similar way to enteric viral pathogens and have similar fates and survival patterns [5]. Monitoring the presence of every specific viral pathogen is impracticable for routine control purposes. Besides the technical difficulties, it would be extremely time-consuming and prohibitively expensive, especially for those countries in most urgent need of efficient water quality control. Therefore, easy-to-detect bacteriophages have been proposed as indicators of fecal and viral pollution and are now included in multiple water quality regulations and guidelines worldwide [6].

Presented here is a general overview of the potential of bacteriophages as fecal pollution indicators, not only in water environments, but also in a range of solid matrices.

## 2. Families of Bacteriophages Used as Indicators of Fecal Pollution

Bacteriophages capable of infecting enteric bacteria are generally classified into three taxonomically diverse groups: somatic coliphages, F-specific coliphages, and bacterio-phages capable of infecting *Bacteroides* spp. [7,8]. Though less common, enterophages (bacteriophages capable of infecting *Enterococcus* spp.) also have valuable potential as indicator organisms due to their high concentrations in wastewater, similar survival rates to enteric viruses, and differential prevalence in human or animal gut microbiota [9,10]. Nevertheless, their suitability has only been tested in tropical regions and further studies are necessary [5,11].

Somatic coliphages are a heterogeneous group of bacteriophages capable of infecting *E. coli* and other coliform bacteria through the cell wall after becoming attached to specific receptors on the outer membrane [12]. Under optimal physiological conditions, lysis occurs approximately 30 min after attachment, and between 100 and 1000 of progeny are released per infected cell [13]. Four major families of somatic coliphages have been described in polluted wastewaters; the most abundant are the *Myoviridae* and *Siphoviridae*, followed by the *Podoviridae* and *Microviridae* [14]. The phage families differ in morphology and resistance to inactivation factors. The *Microviridae* phages differ genetically from the other three families in having single-stranded (ss) rather than double-stranded (ds) DNA. *Microviridae* phages have tailless isometric capsids of 25–30 nm; those of the *Myoviridae* family have capsids of up to 100 nm and a long contractile tail; the isometric capsids of *Siphoviridae* phages are up to 60 nm and have long non-contractile tails; and *Podoviridae* phages have isometric capsids of up to 65 nm with short tails. The somatic coliphages most commonly used as model organisms in research are ΦX174, T2, and T7 [8].

Due to the availability of new genetic data, phage classification by the ICTV is currently undergoing a major overhaul, with new families being described and existing ones divided. Following this reclassification, bacteriophages capable of infecting *E. coli* from the *Caudovirales* order (dsDNA viruses) now include the new families *Ackermannviridae*, *Autographviridae*, *Chaseviridae*, *Demerecviridae*, and *Drexlerviridae* [15,16]. As the contribution of the recently described families to phage presence in the environment has still not been clearly defined, the initial four groups of somatic coliphages (*Myoviridae*, *Siphoviridae*, *Podoviridae*, and *Microviridae*) [14] remain valid, as they are distinguished on a morphological basis. In contrast, some of the new families, defined by genetic differences, are indistinguishable morphologically (e.g., *Ackermannviridae* and *Chaseviridae* are myophages, *Autographviridae* are podophages, *Drexlerviridae* and *Demerecviridae* are siphophages).

Somatic coliphages are the most abundant group of indicator bacteriophages in almost all environmental samples [7]. They have less resistance to disinfectants such as UV light than other bacteriophages, but more than bacterial indicators [13]. Their potential replication in environments outside the gut was once a concern [17,18], but has proved to be negligible for several reasons: their narrow host range; the high concentrations of host and phages required; the possible interference of accompanying microbiota and other particles with the replicative process; the low metabolic activity of hosts in environmental conditions; and responses to environmental stresses possibly minimize phage infection [19,20,21]. It has also been proven that neither coliphages isolated from wastewater nor laboratory stock coliphages are capable of replicating in natural samples, even in tropical climates with more conducive temperatures. It can therefore be concluded that the proportion of somatic coliphages in a natural sample arising from replication in the environment will be practically null [4,22].

F-specific bacteriophages are the second most widespread indicator phages in the environment [7]. They are capable of infecting *E. coli* and other coliform bacteria through the sexual pili, encoded in the F-plasmid, which is transferable to enteric bacteria via conjugation [23]. This group includes two phage families: the *Leviviridae*, ssRNA phages with isometric tailless capsids of around 25 nm, and the *Inoviridae*, ssDNA phages with flexible filamentous capsids of 800 nm [24]. The use of RNase in culture methods permits the differentiation between F-DNA (*Inoviridae*) and F-RNA (*Leviviridae*) coliphages. Based on nucleotide analysis, the *Leviviridae* are divided into two genera, *Levivirus* and *Allolevirus* [25], which include four different genotypes of F-RNA phages: those of subgroups I and II belong to *Levivirus* and subgroups III and IV belong to *Allolevirus* genera [26,27]. Model coliphages representing these groups are MS2 and f2 from genotype I, GA from genotype II, Qβ from genotype III, and FI from genotype IV. The study of these different subgroups is particularly useful for identifying the origin of fecal contamination, since genotypes I and IV predominate in waters contaminated by animal residues whereas genotypes II and III are commonly associated with human contamination [28], though these associations do not always hold, and cross-reactivity has been recorded [29]. F-RNA phages are typically more abundant than F-DNA phages (for instance, 90–95% of F-specific coliphages in wastewater are F-RNA phages) and morphologically more similar to enteric viruses [30,31]. The replication of F-specific bacteriophages outside the gut is considered extremely improbable, as sexual pili cannot be synthesized under 32 °C [32].

F-specific bacteriophages can perform more accurately as indicators in samples where they predominate, such as groundwater, clay sediments, and reclaimed waters; they are also useful for monitoring water treatments such as UV disinfection [33,34,35]. In contrast, they have a low persistence in surface waters, especially in warmer climates, and are readily inactivated by heat or high pH [13]. Therefore, a combination of both types of coliphages may be preferable in some types of samples. Total coliphages can be determined by either summing the results of somatic and F-specific coliphage detection assays or using a host strain that determines both in only one assay [36].

The third group of bacteriophages proposed as indicators infect *Bacteroides* spp. and their concentrations in feces or fecally contaminated samples are usually lower compared to coliphages [37]. Most of these morphologically homogenous bacteriophages belong to the *Siphoviridae* family and infect bacteria through receptors in the cell wall [38,39]. They have a narrow host range, with a high specificity for the cell wall receptors of a particular host [40]. Infectivity seems to be limited by the amount of bacterial capsules, which hamper phage access to the receptors [41]. *Bacteroides* spp. strains are commonly used for microbial source tracking (MST) as they are strongly associated with a given human or animal host and differ in their capacity to recover phages from samples fecally contaminated by different species [41,42]. In general, their utility as MST markers also depends on the geographical location [37,43]. For example, in southern Europe, strain *B. thetaiotaomicron* GA17 can detect phages of human fecal origin, unlike other strains such as *B. fragilis* RYC2056 or HB13 [44] yet in the UK, a *B. fragilis* strain (GB-124) isolated in Brighton [45] was more efficient in this respect. Other examples of geographical variability are strains *B. fragilis* HSP40 [42], *B. fragilis* HB13 [46], and *B. thetaiotaomicron* ARABA 84 [47].

Other strains such as *B. thetaiotaomicron* CW18, *B. fragilis* PG76, PL122, and PZ8 have been isolated and used to detect phages as markers of bovine, porcine, and aviary fecal contamination [48,49]. Despite their low concentrations in water, *Bacteroides*-infecting phages are more resistant to most inactivating factors and treatments than coliphages. Their replication outside the gut is even more improbable, as the host strains are strictly anaerobic and require specific nutrients, such as hemin, that are scarcely found in the environment [42].

Metagenomic studies using sequences from fecal samples available in databases discovered the most abundant phage in the human fecal virome, named crAssphage (cross assembly phage) [50]. Sequence similarities pointed to a group of *Bacteroides*-infecting phages with short non-contractile tails from the *Podoviridae* family [50,51], later confirmed by isolation through culture methods. The morphology of the first isolated crAssphage in *B. intestinalis*, ΦcrAss001, was compatible with *Podoviridae* viruses [52]. One of the main characteristics of ΦcrAss001 is its peculiar replicative cycle; although seemingly a virulent bacteriophage, it can coexist in apparent equilibrium with its host without causing cell lysis, which might benefit both bacteria and virus in a strongly competitive environment like the gastrointestinal tract. The recently isolated crAssphage species such as ΦcrAss002 seem to follow a similar replicative pattern. Efforts to obtain lysogenic ΦcrAss001 have failed so far, though other crAssphage have integrases compatible with lysogenic cycles in their genome [53]. Therefore, expanding our knowledge about crAssphage replication could help to promote their use as fecal indicators with culture techniques [54,55]. CrAssphage have potential application as MST markers, being highly specific to humans and having an extensive geographical distribution and no seasonal variation. They are abundantly detected in human feces (constituting about 90% of the human gut virome), and in sewage throughout the year, as well as in mussels and sediments collected in areas contaminated with wastewater [56,57]. Nevertheless, crAssphage have also been found in several animal sources, so further research is required on possible animal-associated variants or specific genome regions more suitable for animal source discrimination [58,59]. They also have stronger environmental persistence than bacteria and higher concentrations than enteric viruses in sewage worldwide, allowing a more accurate description of virus removal [60,61,62]. These characteristics make crAssphage a very promising alternative as indicator microorganisms of viral fecal pollution, which could be used in MST for monitoring human fecal pollution of water [63,64,65].

## 3. Methods to Detect Bacteriophages

Strategies for detecting phages in samples can be culture-dependent or molecular, each with its own advantages, disadvantages, and appropriate applications.

### 3.1. Culture-Dependent Methods

Culture-dependent methods, available since phages were first discovered, provide qualitative or quantitative information about infectious phages in samples [66]. These methods have already been registered as standardized protocols, mainly by two regulatory bodies: the International Standardization Organization (ISO) and the United States Environment Protection Agency (Washington, DC, USA, U.S. EPA). The ISO provides standardized methodologies for detecting somatic [67], F-specific [68], and *Bacteroides*-infecting phages [69] (ISO 10705), each of which includes two different approaches: a spot test (a qualitative presence/absence protocol that can be adapted to quantitative results using a most probable number approximation) and a double agar layer (DAL) assay (a quantitative protocol for counting plaque-forming units (PFU) in samples). ISO methods can be easily implemented in routine microbiology laboratories without previous experience in working with phages [70], and provide optional steps for laboratories with limited equipment, and quality control assays. U.S. EPA standardized protocols for detecting somatic and F-specific coliphages [71,72] also include two different methods compatible with both types of coliphages: Method 1601 (a quantitative method based on single agar layer (SAL) assays for PFU enumeration) and Method 1602 (a qualitative method based on presence/absence assays), both of which have been successfully validated, have simplified versions [73,74,75], and have been recently revised in Methods 1642 and 1643 [76,77]. Due to the lack of a specific standardized protocol, total coliphage detection is performed using methodologies for F-specific coliphages [36].

ISO and U.S. EPA employ different host strains for the targeted phages, but their equivalent protocols usually give similar counts [36,78,79]. Host strains derived from *E. coli* C are reported to provide the highest counts of somatic coliphages [80]. Both regulatory bodies use nalidixic acid-resistant variants of this strain: *E. coli* CN13 (BCRC17137, ATCC 700609) in U.S. EPA, and *E. coli* WG5 (CIP 107680, ATCC 700078) in ISO methods [67,71,72]. Nalidixic acid-resistant strains were selected to minimize the growth of accompanying microbiota, which frequently interfere with the visualization of plaques. Otherwise, a previous filtration step is required, using membrane filters of 0.22 μm pore diameter made of materials that do not adsorb proteins. To detect F-specific coliphages, host strains must express the sexual pili, encoded in the F plasmid or F-derived plasmids; those in current use are *Salmonella enterica* WG49 (NCTC 12484, CECT 4625, ATCC 700730) [81] and *E. coli* HS/FAmp (ATCC 700891) [82] in ISO and U.S. EPA methods, respectively. Both strains have markers for improving strain selection and stability: Ampicilin resistance (*E. coli* HS/Famp) and lactose degradation capacity (*S. enterica* WG49) [68,71,72].

Host strains initially proposed for the F-specific protocols could also detect somatic coliphages and were suggested for the monitoring of total coliphages, although a standardized culture method has not been described. More efficient strains have been subsequently developed for this purpose: *E. coli* C3000 (ATCC 15597), which is mainly used in the U.S., detects lower amounts of somatic coliphages than the standardized strains, and *E. coli* CB390 (CECT9198), which can recover both groups of coliphages with similar efficiency to its standardized counterparts [36,83]. The standardized method for detecting *Bacteroides*-infecting bacteriophages uses *B. fragilis* RYC2056 (ATCC 700786) as a host strain, although other strains can be employed to discriminate between human and animal fecal pollution [69,84].

Standardized culture methods are simple, robust, cost-effective, and easily prepared, especially for coliphages, which do not require anaerobic growth conditions. The methods can be scaled to different sample volumes, maintaining the same proportions between medium, host strain, and sample. The material, media, reagents and labor have a similar cost to the methods currently used in routine analysis laboratories to detect fecal coliforms/*E. coli*. Costs may increase by 10–15% if an additional step with RNAase is required for the recovery of F-RNA and F-DNA coliphage subgroups, or due to the longer incubation times required for anaerobic *Bacteroides* spp. [8]. The standard methods could be optimized further to improve phage recovery and the cost/benefit relationship; modifications could include diluting the medium concentration, substituting components, and optimizing incubation protocols [79,85].

In general, standardized methods are time-consuming, requiring more than one working day to obtain reliable results (at least 18 h for coliphage plaques and more than 48 h for *Bacteroides* bacteriophages). To prevent potentially virally contaminated water being used for consumption, irrigation, or recreation [86,87], the results need to be obtained on the same day as the analysis. Shorter operative times and incubation periods, as well as more user-friendly handling, are also warranted by the increasing implementation of coliphages in guidelines and regulations. Several modified standardized methods have already been developed in this respect [88], the most promising being Easyphage and Quantiphage, which incorporate non-agar-based supports for plaque counting and previously prepared components for greater speed and simplicity [89,90]. Other promising modifications are based on the detection of enzymatic lysis in liquid cultures, focusing primarily on the activity of β-galactosidase [91], adenylate kinase [92], and β-glucuronidase [93]. Among these promising fast methodologies, three have commercial application: Fastphage (already validated in U.S. EPA methods), Quantiphage and Bluephage (currently in development). Fastphage and Bluephage use the activity of a liberated intracellular enzyme as an indicator of cell lysis (β-galactosidase and β-glucuronidase, respectively), the presence/absence of phages indicated by color changes, while Quantiphage incorporates cellulose supports to achieve a more rapid plaque detection [88].

### 3.2. Molecular Methods

Molecular methods, although fast and sensitive, have a major drawback in that they cannot provide information about infectivity, which therefore requires additional steps. Without infectivity data, viral concentration, and human health risks are often overestimated [94]. Molecular methods can be serological or involve nucleic acid-based or microelectronic sensors.

Serological techniques are rapid and can be applied in situ, but they require pre-enrichments, and antisera are less available than nucleic acid probes and primers [8]. They are mainly applied to detect F-specific coliphages, using latex agglutination or neutralization methods [95], being less suitable for the highly diverse and complex somatic coliphages, though CLAT- (Culture, Latex Agglutination, and Typing) based analyses have been developed for some specific families [96].

Nucleic acid methods are based on plaque hybridization, employing specific probes or, more frequently, qPCR/RTqPCR assays. They are mainly used to detect F-specific and *Bacteroides*-infecting phages [8], and, as with serological methods, have limited applications for somatic coliphages, though PCR and qPCR techniques have been developed for specific families or bacteriophages [96]. In plaque hybridization methods, specific probes designed for each targeted phage or phage group are applied to plaques obtained by culture [26]. RT-qPCR analyses are used to quantify the number of genome copies (GC) present in a given sample. The quantities detected by molecular methods tend to be higher compared with culture methods, as GC signals are more persistent in the environment and more resistant to treatments than infectious viruses [29]. In order to solve this discrepancy, nucleic acid amplification techniques based on the membrane or capsid integrity have been developed. However, membrane integrity does not equate with viability and therefore cannot serve as a control of the efficacy of inactivation mechanisms that do not directly target cell membranes [97,98]. PCR-based approaches can also be inhibited by organic substances such as phenolic compounds, which are occasionally present in environmental samples [99]. Molecular methods are available for F-specific coliphage detection, but solely for genogroups of F-RNA-specific coliphages or specific phages such as MS2 [27,100]. PCR-based methods have also been developed to detect certain *Bacteroides* phages, facilitating the recovery of phages associated with a certain animal host [101]. As demonstrated by the discovery of crAssphage, metagenomics studies of the gut bacteria open the possibility of identifying sequences of new bacteriophages that infect non-cultivable host bacteria that are specific of certain species [37]. When used as fecal indicators, crAssphage are predominantly detected and quantified in environmental samples by qPCR assays [58,102,103]. Their isolation from environmental samples by lysis plaque formation using DAL is still difficult, due to the absence of appropriate hosts and a still unexplored biological replicative cycle [61]. CrAssphage are also suitable for fecal source discrimination and have been shown in comparative MST molecular qPCR assays to have advantages over existing bacterial markers (such as HF183/BacR287) in terms of specificity, accuracy and high sensitivity [58,102,103].

Microelectronic methods involve the detection of viral particles, or the lysis of host bacteria caused by bacteriophage infection [88]. Although fast, with results being obtained in less than 1 h, their sensitivity and precision do not yet match DAL-based methods [104,105,106]. Their usage is normally restricted to the detection of a specific phage, rather than to analyze environmental samples containing different phages at varying concentrations. Consequently, no microelectronic method so far has achieved a useful or feasible application to determine infectious bacteriophages as indicators of fecal pollution in environmental samples.

Viral concentration methods to optimize detection processes have been developed in parallel with standardized methodologies. For the analysis of larger volumes, the samples need to be concentrated, especially if quantification is required, as in drinking water samples with low levels of contamination. Two methods are recommended to concentrate volumes of up to 1000 mL, depending on the turbidity of the sample. When turbidity is low, a simple, inexpensive, and practical procedure is recommended, using mixed cellulose and acetate membrane filters with a pore size of 0.45 μm after the addition of salts and pH adjustment [107,108]. When turbidity is high, flocculation with magnesium hydroxide is feasible for all three groups of fecal indicator phages [109,110]. Furthermore, phages can also be concentrated by ultrafiltration, like other viruses [8].

## 4. Application of Bacteriophages as Indicators of Fecal Pollution

### 4.1. Bacteriophages as Fecal Indicators in Water

As already mentioned, FIB are used to estimate the microbiological quality of water, but they may not be suitable or sufficiently reliable to predict the presence of enteric viruses. In general, enteric viruses have higher survival rates during wastewater and drinking water treatment than bacterial indicators and greater persistence in environmental waters [17,34,110,111,112]. Therefore, the use of bacterial indicators alone could underestimate the microbiological contamination of water and the associated human health risks. Adding at least one viral indicator to the analysis provides a more accurate assessment of water quality and promotes more confidence in its safety.

**Figure 1:**
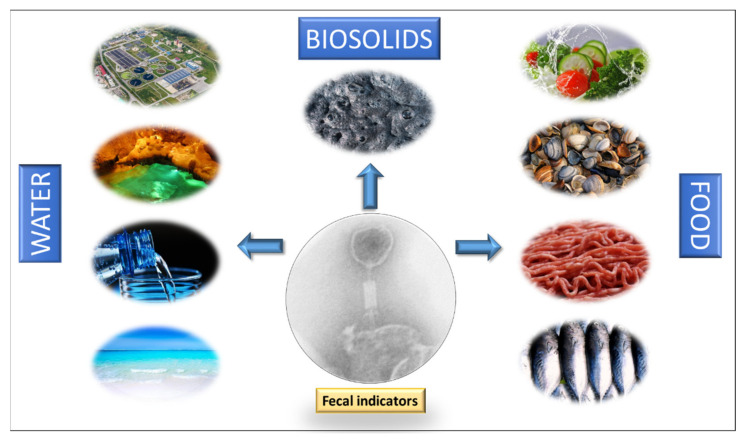
Fields of application of bacteriophages as fecal indicators.

The primary origin of coliphages in water environments are human and animal feces. They can reach water through raw or treated human and animal wastewater, septic tank overflow, sewer leakage, and the spread of solid waste (sewage sludge, slurry, manure, and the feces of pets, farmed animals, and wild animals) [7]. As early as 1948, Guelin [113] already saw the potential of coliphages as indicators of enteric microorganisms in water, observing their good correlation with the numbers of coliform bacteria in fresh and marine water. Since then, many other studies have assessed the potential of bacteriophages as indicators of fecal contamination in different water environments (Figure 1):

Wastewater treatment plants: Bacteriophages are considered to be useful tools to evaluate the efficacy of wastewater treatment plants [112,114,115,116] because their reduction by certain pathogen removal methods is similar to that of human enteric viruses [114,117] whereas the reduction of traditional FIB is significantly higher [5]. Coliphage concentration in wastewater shows no seasonality and remains consistently high throughout the year worldwide, as occurs with bacterial indicators [114,118,119]. Coliphage densities in wastewater are quite variable but the lower density of F-specific coliphages compared to somatic coliphages in both treated and untreated wastewater sources [120], potentially limits their use as indicators in this environment.

Drinking water: The presence of coliphages or *Bacteroides*-infecting phages in drinking water sources is a likely indicator of fecal contamination or an inadequate treatment [121]. Generally, the levels of bacterial indicators, viruses, and bacteriophages in these sources are low and seldom detected after treatment. The few reports describing the potential of phages to assess the quality of drinking water suggest once again that they outperform conventional FIB, undergoing less reduction after different drinking water treatments [122,123,124,125,126].

Recreational water: The sanitary quality of recreational waters is monitored using FIB according to the EU Directive 2006/7/EC [127], but alternative indicators, such as *Clostridium perfringens* and bacteriophages, have also been proposed [111]. Somatic coliphages are found in recreational water [128], and at beaches with unknown sources of fecal contamination, the presence of coliphages correlates with the occurrence of diseases more often than the presence of FIB [129,130]. It has been reported that in waters with detectable coliphages there was an increased incidence of gastrointestinal illness among bathers when fecal pollution was likely present, but not otherwise [131]. Compared with enterococci, the correlation was similar for somatic coliphages and even higher for F-specific coliphages [131]. These findings indicate that coliphages may be suitable for application as indicators of bathing water quality.

Groundwater: Groundwater constitutes an important fraction of the water used for household and municipal supplies, agriculture and landscape irrigation, and industry. Contaminants reaching surface waters also affect groundwater, the routes including failure in septic systems, leaking sewer lines and passage through soils and fissures. A study shows that one bacterial indicator and one phage indicator provide more information than two bacterial indicators when assessing the microbiological quality of groundwater [34]. However, as shown in Table 1, to date, only one regulation (2006) includes bacteriophages as indicators of enteric viral pollution in groundwater, reflecting that further research is needed in this area.

### 4.2. Bacteriophages as Fecal Indicators in Solid Matrices

Solid or semisolid matrices play an important role in the persistence and dispersion of pathogens in the water cycle, as they can contain large amounts of pathogens, especially viruses, if contaminated with fecal waste [6,145].

To optimize the use of bacteriophages as indicators in solid matrices (Figure 1), standardized methodologies for their extraction, detection, and enumeration need to be developed. The current methods differ according to the matrix, which hinders the comparison of results. Nevertheless, in general, studies indicate that somatic coliphages are found in solid matrices at higher levels than traditional FIB and F-specific RNA coliphages. In addition, they persist longer in soils and sediments and are more resistant to sludge and manure treatments. For an extensive review of this question, and more data on solid matrices, see Martín-Díaz et al. [6].

### 4.3. Bacteriophages as Fecal Indicators in Food

Coliphages can be found in food when fecally contaminated water is used to grow vegetables and fruits, in meat processing, or to farm shellfish. Bivalve shellfish are regularly implicated in foodborne viral disease outbreaks because there is no effective way to rid them of viral contamination without changing their sensory characteristics. Instead, efforts are focused on preventing contamination. Shellfish accumulate and concentrate bacteriophages and viruses through their feeding process, and their depuration systems are more efficient for eliminating bacteria than viruses. Accordingly, Blanco-Picazo et al. found somatic coliphages in 70% of the tested shellfish samples but no *E. coli* [146]. However, the utility of phages as routine indicators of viral pollution in shellfish, in contaminated sites, or under normal growing conditions, is controversial; studies with conflicting results have been reviewed [147]. Regardless, the use of bacterial indicators alone is clearly insufficient to prevent viral disease outbreaks stemming from shellfish consumption [148] and more accurate data about enteric viruses could be provided by the addition of a viral indicator.

Regarding fish, a comparative study found somatic coliphages in Atlantic, farmed and frozen fish, with 30%, 10%, and 20% of the samples testing positive, respectively; in contrast *E. coli* was only found in 10% of the Atlantic fish samples [146].

Enteric bacteriophages have been proposed as potential fecal indicators in different types of meat. Hsu et al. found somatic coliphages in 88% of ground meat and poultry meat samples and F-specific coliphages in 63%. They also evaluated the risk of fecal contamination at three control points (evisceration, washing and chilling) and observed that the reduction of F-specific coliphages during these processing steps matched that of FIB [149]. Somatic coliphages have also been reported in minced pork, minced chicken, and ham, with 60%, 100%, and 40% of the samples testing positive, respectively. No *E. coli* was found in ham samples, and only 30% of minced pork and 90% of minced chicken samples tested positive for this bacterial indicator [150].

In a study of animal feeds, Maciorowski et al. analyzed animal feeds, feed ingredients, and poultry diets for the presence of coliphages, finding somatic and F-specific coliphages in all the tested samples, even after 14 months of storage at −20 °C [151].

Bacteriophages can also be used as fecal indicators in vegetables, as they have been found in lettuce and cucumber [152]. In lettuce, the number of samples positive for *E. coli* was slightly higher compared to somatic coliphages (50% and 40%, respectively); however, 20% of cucumber samples were positive for coliphages and none contained *E. coli* [152].

All these data suggest bacteriophages perform well as fecal indicators in food (Figure 1) as they seem to remain longer in the different food matrices than bacteria.

## 5. Regulations and Future Perspectives

Bacteriophages, specifically coliphages, have been included as viral indicators of fecal pollution in several water quality policies and guidelines over the last two decades, as bacterial indicators have proven to be ineffective for predicting viral outbreaks in water and food samples [153]. It is of particular importance that EU regulations for drinking and reclaimed water have recently incorporated coliphages as parameters of microbial quality. [132,133]. The number of regulations including bacteriophages can therefore be expected to increase dramatically in the next decade, after member states of the EU adopt this legislation. The current regulations and guidelines around the whole that include bacteriophages as indicator organisms can be found in Table 1. Moreover, a consequence of the COVID-19 pandemic is that interest in the control of viral contamination is likely to increase, due to health concerns and the growing public awareness of viral infection, though SARS-CoV2 is not a waterborne pathogen [154,155].

The inclusion of coliphages in regulations means that standardized techniques for their enumeration need to be improved and optimized, to enable faster and simpler testing. Considering the ongoing research in this field, it seems likely that streamlined user-friendly kits providing results in a few hours at very reasonable costs will become available in the near future [83].

Regarding crAssphage, research is expected to grow in the coming years as new phages from this family are isolated and host strains are described [48,49]. Studies of particular interest will be focusing on the replication cycle of crAss-like bacteriophages, their high persistence in the human gut microbiota, their prevalence in wastewater and other aquatic environments, their significance for human intestinal physiology and disease, and the development of culture techniques. Evidence from this research will help to elucidate the true value and suitability of crAssphage as a fecal indicator and MST marker.

## 6. Conclusions

Bacteriophages are attractive as promising alternative fecal indicators to assess the risk of viral contamination in natural and built environments. Further research is needed to facilitate their application, including the development of improved standardized methods, but there is no doubt that they are already a valuable complement to existing methodologies.

## Figures and Tables

**Table 1:** Current guidelines and regulations around the globe that include bacteriophages as indicators of fecal pollution [132,133,134,135,136,137,138,139,140,141,142,143,144].

Country/Organization	Biosolids	Ground Water	Recreational Water	Drinking Water	Reclaimed Water	Membrane Integrity & UV	Direct Potable Reuse
Australia	2012 (WA)	–	–	2011 *	2005/2011 (QL/WA)	–	–
Canada	–	–	–	2011 (Q)	–	–	–
Colombia	2014	–	–	–	–	–	–
EU	–	–	–	2020	2020	–	–
India	–	–	–	2012	–	–	–
Singapore	–	–	–	2017 * (WHO)	–	–	2017 * (WHO)
South Africa	–	–	–	1996 *	–	–	–
USA	–	2006	2015 (Prop)	–	2011 (NC)	2015	–
WHO	–	–	–	2017 *	2017 (I&AR)	–	2017 *

WA: Western Australia, QL: Queensland, Q: Quebec, NC: North Carolina, Prop: Proposal, I&AR: Irrigation and aquifer recharge, * Guideline.
